# Supplementary material for: Activating Telomerase TERT Promoter Mutations and Their Application for the Detection of Bladder Cancer
Source: Int J Mol Sci. 2020 Aug 21;21(17):6034. doi: 10.3390/ijms21176034 (PMC7503716; doi:10.3390/ijms21176034)
Supplement: Supplementary file 1 [file ijms-21-06034-s001.pdf]

Supplementary

Additional description of table 1

To simulate possible structures of DNA near the two TERT-promoter mutations we picked up the region which covers almost all fragments used for amplification in the vast majority of described research. The most appropriate fragment, which must have an optimal length i.e., include only closest G-quadruplexes susceptible to influence of point mutations, was used in the study [1]. G strands of 3 types of DNA (WT, only with mutation C228T and only with mutation C250T) were analyzed by web-server "QGRS-mapper" (http://bioinformatics.ramapo.edu/QGRS/index.php) with the following parameters: max length = 30 bases, min G-group = 2, loop size 0–36 [2]. Whole C-strand was also analyzed by "QGRS-mapper" but this analysis didn't reveal any G-quadruplexes. Regions of C-strands, which are complementary to G-quadrpulexes of G-strand, were analyzed by "ViennaRNA Web Service" (http://rna.tbi.univie.ac.at/) to reveal possible loops. The colored figure was created using the web-based server "Biorender" (https://biorender.com/). As a result, G-strand always contained 6 G-quadruplexes, whose structures (e.g., number of G-tetrads) and positions altered depending on the type of sequence. C-strand, in turn, always contained 2 loops in the complementary regions to G-quadruplexes.

Table S1. G-quadruplexes of WT G-strand.

Position	Length	QGRS	G-Score
53	19	**GGAGAGGGCGGGGCCGCGG**	20
80	17	**GGGAGGGGCTGGGAGGG**	41
100	17	**GGAGGGGGCTGGGCCGG**	21
123	18	**GGGAGGGGTCGGGACGGG**	42
154	11	**GGAGGAGGCGG**	21
173	19	**GGTGAAGGGGCAGGACGGG**	20

Table S2. G-quadruplexes of G-strand with C228T.

Position	Length	QGRS	G-Score
59	18	**GGCGGGGCCGCGGAAAGG**	20
80	17	**GGGAGGGGCTGGGAGGG**	41
104	22	**GGGGCTGGGCCGGGGACCCGGG**	41
128	18	**GGGTCGGGACGGGGCGGG**	42
154	11	**GGAGGAGGCGG**	21
173	19	**GGTGAAGGGGCAGGACGGG**	20

Table S3. G-quadruplexes of G-strand with C250T.

Position	Length	QGRS	G-Score
59	18	**GGCGGGGCCGCGGAAAGG**	20
80	17	**GGGAGGGGCTGGGAGGG**	41
105	20	**GGGCTGGGCCGGGGACCCGG**	21
128	18	**GGGTCGGGACGGGGCGGG**	42
154	11	**GGAGGAGGCGG**	21

173	19	**GGTGAAGGGGCAGGACGGG**	20

Table S4. Applicability of different test systems to mutation analysis in patients with hematuria.

Article	Cohort Size	Number of Patients with Hematuria	Number of TERT-Positive Patients
[3]	475 (Gross hematuria)	475<br>99 with BC<br>376 without BC	81 (sensitivity of 81,8%)<br>314 (specificity of 83,5%)
[4]	234 (BC and control group with hematuria)	234<br>135 with BC (no information on hematuria)<br>39 without BC	-<br>4 (specificity of 90%)
[5]	570 (Early detection cohort; Hematuria or LUTS)	509<br>163 with BC<br>346 without BC	90 (sensitivity of 55%)<br>15 (specificity of 96%)
[6]	125 (BC and control group with hematuria)	125<br>92 with BC (Supernatant-urine cfDNA)<br>33 without BC (Supernatant-urine cellDNA)<br>92 with BC (Sediment-urine cellDNA)<br>33 without BC (Sediment-urine cellDNA)	46 (sensitivity of 50%)<br>0 (specificity of 100%)<br>48 (sensitivity of 52%)<br>0 (specificity of 100%)

LUTS: Lower Urinary Tract Syndrome.

References

1. Wang, K., et al., TERT promoter mutations are associated with distant metastases in upper tract urothelial carcinomas and serve as urinary biomarkers detected by a sensitive cast *PCR*. Oncotarget, **2014**. *5* 12428–39.
2. Kikin, O., L. D'Antonio, and P.S. Bagga, QGRS Mapper: a web-based server for predicting G-quadruplexes in nucleotide sequences. *Nucleic Acids Research*, **2006**. *34*(suppl_2): W676–W682.
3. Dahmcke, C.M., et al., A Prospective Blinded Evaluation of Urine-DNA Testing for Detection of Urothelial Bladder Carcinoma in Patients with Gross Hematuria Eur. Urol., 2016. **70**, 916–919.
4. Allory, Y., et al., Telomerase reverse transcriptase promoter mutations in bladder cancer: high frequency across stages, detection in urine, and lack of association with outcome Eur. Urol., 2014. **65**(2): p. 360–366.
5. Springer, S.U., et al., Non-invasive detection of urothelial cancer through the analysis of driver gene mutations and aneuploidy. *Elife*, **2018**. *7*.

6. Ou, Z., et al., Detection of bladder cancer using urinary cell-free DNA and cellular, *DNA. Clinical and Translational Medicine*, **2020**. *9*, e4.
